# The Efficacy of Jing Wan Hong Ointment for Nerve Injury Diabetic Foot Ulcer and Its Mechanisms

**DOI:** 10.1155/2014/259412

**Published:** 2014-11-16

**Authors:** Shumei Jin, Mixia Zhang, Yan Gao, Xuebin Zhang, Guangzhi Cui, Yanjun Zhang

**Affiliations:** ^1^Tianjin Huanhu Hospital, Tianjin 300060, China; ^2^Chinese Materia Medica College, Tianjin University of Traditional Chinese Medicine, No. 312 Anshanxi Road, Nankai District, Tianjin 300193, China; ^3^Tianjin State Key Laboratory of Modern Chinese Medicine, Tianjin University of Traditional Chinese Medicine, Tianjin 300193, China; ^4^Institute of Traditional Chinese Medicine, Tianjin University of Traditional Chinese Medicine, Tianjin 300193, China; ^5^Tianjin Children's Hospital, Tianjin 300074, China

## Abstract

Jing Wan Hong ointment contains 30 kinds of Chinese herbs, with functions of activating blood circulation to disperse blood stasis, clearing heat, eliminating dampness, and reducing swelling by detoxification. Therefore, Jing Wan Hong ointment may facilitate the healing of ulcers. The aim of this study was to evaluate the efficacy and mechanisms of Jing Wan Hong ointment for healing diabetic foot ulceration in Wistar rats induced by streptozotocin and sciatic nerve damage. The results showed that Jing Wan Hong ointment had a marked effect on foot ulcers in diabetic rats induced by initial nerve injury. These effects were manifested by reducing the foot ulcer size and Wagner grade after seven days of treatment. The diabetic rats with foot ulcers were almost healed after 21 days of treatment. Moreover, the mechanisms of this effect seem to be dependent on increased expression of PDGF mRNA, but there was no influence on the expression of TGF-*β*, VEGF, and FLT-1 mRNA.

## 1. Introduction

The diabetic foot is a chronic complication of diabetes mellitus, characterized by infection, ulceration, or destruction of deep tissues, associated with neurological disorders in various degrees of peripheral vascular disease of the lower limbs. Worldwide prevalence of diabetes among adults (aged 20–79 years) was 6.4%, affecting 285 million adults, in 2010, and will increase to 7.7%, affecting 439 million adults, by 2030 [1]. Diabetic foot thus has a substantial social and economic impact, including disabilities affecting employment and high costs associated with its control and treatment, in addition to its acute and chronic complications [2].

Diabetic foot ulceration (DFU) results in the high risk of lower extremity amputation due to the damage of both macro- and microvascular neuropathy. Peripheral neuropathy is a devastating complication of diabetes mellitus, affecting up to 50% of those afflicted with this disease [3]. Diabetic foot ulcer or gangrene is one of the most serious complications and, therefore, it is a particular concern in current research. Traditional Chinese medicine (TCM) has been widely accepted as an alternative to or in combination with conventional medicine for treating different kinds of diseases. Jing Wan Hong ointment comprises 30 kinds of Chinese herbs, which mainly include radix ampelopsis,* Angelica dahurica*, Chinese lobelia, borneol, rhizoma atractylodis, red peony root, Rhizoma Ligustici Chuanxiong, and pangolin. It has the function of activating blood circulation to dissipate blood stasis, clearing heat, eliminating dampness, and reducing swelling by detoxification.

In this study, we investigated the efficacy of traditional Chinese medicine Jing Wan Hong ointment for its potential ulcer-healing effects and its mechanisms of wound healing in DFU of Wistar rats induced by sciatic nerve damage, in order to provide reliable experimental evidence for the treatment of neurodegenerative type DFU.

## 2. Materials and Methods

### 2.1. Experimental Animals

Male Wistar rats (200~220 g) were purchased from Tian Jin Shan Chuan Hong Experimental Animal Technology Company (SCXK, 2009-0001, NO0003091 (Jin)). They were housed under the conditions of 22–25°C and a 12 h light-dark cycle with standard animal food and constant access to tap water. The experimental study was performed in accordance with national laws of animal handling and with the permission of the local ethical committee.

### 2.2. Animal Models

#### 2.2.1. Diabetes Model

The diabetes model was induced by injecting streptozotocin (STZ, Sigma) intraperitoneally (70 mg/kg) into 60 healthy male Wistar rats for two consecutive days. In all groups, blood sugar was monitored daily by glucometer (One Touch Ultra); after one month those whose blood sugar was more than 16.65 mmol/L were used in the following experiment.

#### 2.2.2. Diabetic Foot Ulcer Model

Some rats from the previously established diabetic model were selected to undergo a sciatic nerve injury procedure for the development of the ulcer model. To induce sciatic nerve injury, the rats were anesthetized with an intraperitoneal injection of 10% chloral hydrate (0.003 mL/g); an incision of approximately 1.5–2 cm was made along the right sciatic nerve of the rats; to separate the sciatic nerve from the subcutaneous muscle layer, the sciatic nerve was completely clamped at approximately 0.5 cm above the bifurcation with medium hemostat pliers for one minute and then finally sutured layer by layer. The day that the rats underwent sciatic nerve injury operation was defined as day 0. Seven days after the sciatic nerve injury, the diabetic rats which developed an ulcer on the right foot were selected for the following experiments.

### 2.3. Grouping and Drug Administration

A total of 60 model male Wistar rats were randomly divided into four groups as follows: diabetic control (DC), diabetic and sciatic nerve injury model (DNIM), Jing Wan Hong ointment treatment (JWH) (Tianjin Darentang Jingwanhong Pharmaceutical Co., Ltd.), and positive control. Recombinant human epidermal growth factor topical solution treatment (rhEGF) (Shenzhen Hua Shengyuan Gene Engineering Co., Ltd., about 4000 IU/10∗10CM2) was administered as a positive control.

Animals which did not develop ulcers after seven days were intended to be rejected at the beginning of the experiment. Animals that had developed ulcers were used to continue in the following experiment. JWH and the rhEGF groups were treated according to the above dose by dressing the wound with gauze which was immersed with JWH ointment or immersed with rhEGF, whereas the DC and DNIM groups were treated with a gauze dressing which was only immersed with physiological saline every morning at 9:00 for 21 days.

### 2.4. Wagner Classification

Excluding the unsuccessful models, one to two rats were selected randomly from each group for pathological study and the other 10 rats were used for Wagner classification. The results of Wagner's classification for diabetic foot ulcer were recorded in each group on days 7, 14, 21, and 28. A double-blind method in Wagner classification was standardized by two experimenters. Then, the mean value was selected. The standardizations are as follows (adopted from Levin and O'Neal [4]):

 Grade-0: high risk foot and no ulceration,
 Grade-1: superficial ulcer,
 Grade-2: deep ulcer (cellulitis), not extending to the bone and tendon,
 Grade-3: osteomyelitis with ulceration or abscess,
 Grade-4: gangrenous patches, partial foot gangrene,
 Grade-5: gangrene of entire foot.

Based on the above standardizations, the Wagner's classification was Grade-0 on day 0; thus, the comparison was ignored in the following result.

### 2.5. Measurements of Body Weight and Plasma Glucose

The animals' body weight and plasma glucose were recorded at day 0 and day 28 after administration.

### 2.6. Morphological Observation

#### 2.6.1. Macroscopic Structure

True-color images (24 bits) of the rats' feet were captured using a digital camera (E5200; Nikon, Japan) on day 7 (before administration) and on days 14, 21, and 28 (the 7th, 14th, and 21st day after administration). Digital images were taken from the ulcer positions on the feet of the rats. Later, the foot ulcer morphology in vivo was observed and comparisons were made. The wound area of each rat was also recorded at different times.

#### 2.6.2. Light Microscopy

The foot skin specimens of all groups of rats were partly fixed in 4% paraformaldehyde, dehydrated, and embedded in paraffin for 48 hours. The samples were sliced and then stained with haematoxylin and eosin. The histological structure changes were observed under a light microscope, and color photographs under the image acquisition system (EOS D600) were collected.

### 2.7. RNA Extraction and RT-PCR Analysis

Total RNA was extracted from the ulcer area on day 28 from each group by TRIZOL total RNA extraction kit (Invitrogen) according to the manufacturer's instructions. The RNA concentration was calculated with OD260, and its purity was calculated with OD260/OD280. Moreover, first-strand cDNA was synthesized from 1 *μ*g of total RNA using the Quant cDNA first strand synthesis kit, (Tiangen Biotech (Beijing) Co., Ltd), and reverse transcription (RT) was performed according to the manufacturer's instructions. The cDNAs were subjected to PCR amplification with primers as described in Table 1. The efficiency of the RT-PCR was controlled by glyceraldehyde-3-phosphate dehydrogenase (GAPDH) amplification. The PCR products were subjected to densitometry assay using the Bio Imaging System (Gene Snap) after electrophoresis on a 2% agarose gel and staining with ethidium bromide and data were analyzed with the ratio of the target gene and GAPDH optical density. The DNA Ladder Marker was purchased from Bao Biological Engineering (Dalian) Co., Ltd. (D526A). The primers for PCR are shown in Table 1.

### 2.8. Statistical Analysis

Statistical analysis was performed using the statistical software SPSS20.0. All data were presented as mean ± standard deviation (mean ± SD) and analyzed using one way ANOVA and post hoc least significant difference (LSD) test. General Linear Model Repeated measures and ANOVA was performed in analysis of the wound areas of different groups at different times. Mann-Whitney Rank Sum Test was performed in the analysis of Wagner classification. The value of *P* < 0.05 was considered statistically significant.

## 3. Results

### 3.1. Body Weight and Plasma Glucose

**Figure 1:**
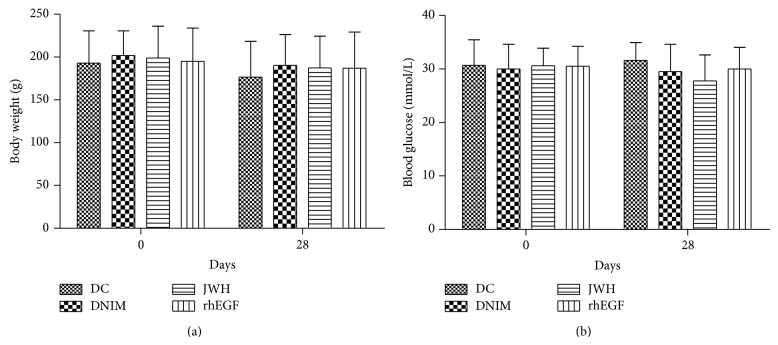
Results of the body weight and plasma glucose of the rats. (a) The body weight on day 0 and day 28 after administration ($\overset{-}{x}\pm S$, *n* = 10). (b) The blood glucose on day 0 and day 28 after administration ($\overset{-}{x}\pm S$, *n* = 10). There are no significant differences between all groups (*P* > 0.05).

After STZ injection, the rats developed typical symptoms of diabetes, and their plasma glucose became more than 16.65 mmol/L. However, there were no significant differences in the body weight and blood glucose levels between all groups on day 0 (*F* = 0.115 or 0.059, *P* > 0.05) and day 28 (*F* = 0.224 or 1.286, *P* > 0.05) after administration as shown in Figure 1.

### 3.2. Wagner Classifications

On day 7, Wagner's classification of all groups of diabetic rats was less than Grade-2; there were significant differences in the other three groups compared with the group DC (*P* < 0.01), but there were no significant differences in these three groups compared with each other (*P* > 0.05). Yet, on days 14, 21, and 28, the Wagner scores were less in the JWH group and rhEGF group than the DNIM group (*P* < 0.05 or *P* < 0.01) as shown in Table 2. In this section, we also compared the JWH group with the rhEGF group; each time there were no significant differences with each other (*P* > 0.05).

### 3.3. Morphological Observation

We observed foot morphology by both macroscopic examination and light microscope in this study. The macroscopic observation results showed that the ulcer treatments were effective in the JWH and rhEGF group. In these two groups, inflammation was alleviated, and the foot ulcers were restituted on the seventh day after administration. Most of them (JWH and rhEGF groups) were almost healed after 21 days of treatment and the percentage of wound closure was above 80%. However, in the DNIM group, the inflammation was increasingly heavy and the foot ulcer was deeply developed into the muscle.

**Figure 2:**
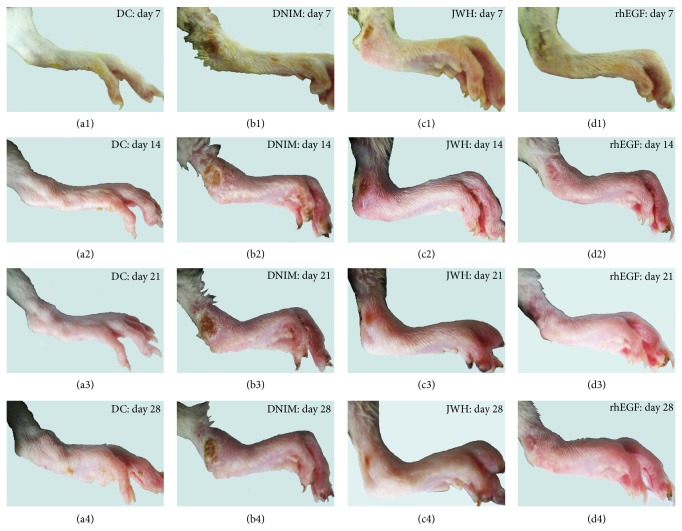
Results of macroscopic observation. These were the photographs of nerve injury diabetic foot of all groups on day 7 (before administration) and days 14, 21, and 28 (after ministration). Each figure was indicated by the foot ulcer. (a1–a4) showed the integral skin of DC group; (b1–b4) showed the foot ulcer changes in DNIM group on days 7, 14, 21, and 28. (c1–c4) showed the foot ulcers forming in the JWH group on day 7 (before administration) and days 14, 21, and 28 (after administration). (d1–d4) showed the foot ulcers forming in the rhEGF group on day 7 (before administration) and days 14, 21, and 28 (after administration).

**Figure 3:**
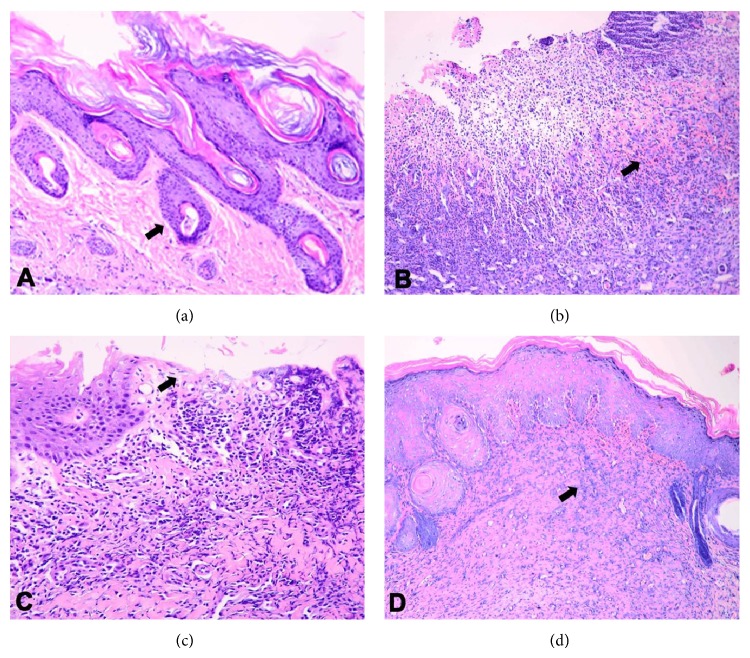
Results of microscopic observation. (a–d): these were the photomicrographs of the skin of each group of nerve injury diabetic foot on day 28. (a) The DC group showed clear skin structure (arrow). (b) The DNIM group showed serious injury and forming ulcer (arrow) deep to the layer of hypodermis. (c) The JWH Group showed the healing ulcer with the regeneration of epidermis (arrow). (d) The rhEGF Group showed the healed ulcer with scarring under the epidermis (arrow) (HE 40x).

The microscopic observation results showed that the skin structure was very clear and flexible in the DC group. While in the DNIM group, the foot skin ulcers formed deeply into the muscle, the inflammatory exudation was profuse, and the necrosis was apparent. However, in the JWH group, the ulcer skins were proximately intact. The inflammatory exudation and necrosis were clear with epidermic cell regeneration. In the rhEGF group, most of the rats' foot skin was healed with a scar under the subcutaneous tissue; the foot skin structures were clear and flexible. Only two of the 10 rats had superficial ulcers extending deep into the corium. All these morphological results coincided with the Wagner's classification of all groups as shown in Figures 2 and 3.

### 3.4. Changes of Ulcer Areas at Different Times

**Figure 4:**
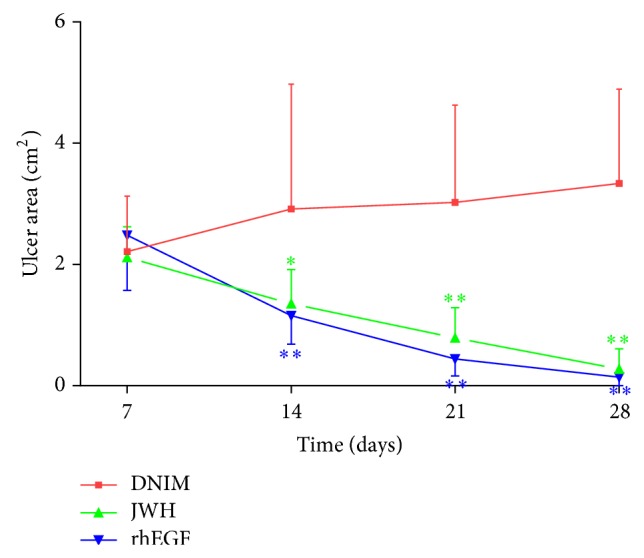
Changes of ulcer area at different times: ulcer areas were indicated with mean ± standard deviation ($\overset{-}{x}\pm S$, *n* = 10). The JWH and rhEGF groups showed that the ulcer area decreased over time (at days 14, 21, and 28). The DNIM group showed the ulcer area increased over time (at days 14, 21, and 28). ^*^ *P* < 0.05 versus DNIM group; ^**^ *P* < 0.001 versus DNIM group.

The diabetic rats of the DC group did not form foot ulcers all the time. Therefore, we have neglected it in comparing the foot ulcer areas. The wound areas of diabetic rats including the DNIM, JWH, and rhEGF groups were calculated at different times and compared with each other on days 7, 14, 21, and 28. The results indicated that the wound areas were significantly changed at different times (*F* = 14.573, *P* < 0.001). It was decreased in the JWH and rhEGF groups, but it was increased in the DNIM group. There are significant differences in different groups (*F* = 15.694, *P* < 0.001). Nevertheless, there was no significant difference between the JWH and rhEGF groups (*P* = 0.836 > 0.05). These changes are shown in Figure 4.

### 3.5. The Effects of Jing Wan Hong Ointment on PDGF, TGF-*β*, VEGF, and FLT-1 mRNA Expression

**Figure 5:**
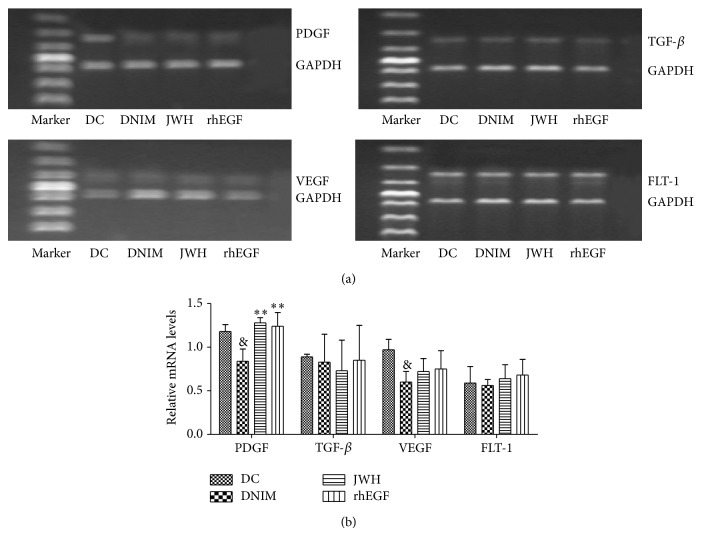
(a) Results of the agarose gel electrophoresis of PDGF, TGF-*β*, VEGF, and FLT-1 mRNA expression. (b) Results of the effects of Jing Wan Hong ointment on PDGF, TGF-*β*, VEGF, and FLT-1 mRNA expression. Values normalized to glyceraldehyde 3-phosphate dehydrogenase (GAPDH) mRNA expression and expressed as mean + SEM ($\overset{-}{x}\pm S$), & denotes *P* < 0.05 versus DC group; ^**^ *P* < 0.01 versus DNIM group.

We analyzed the mRNA expressions of platelet-derived growth factor (PDGF), transforming growth factor-*β* (TGF-*β*), vascular endothelial growth factor (VEGF), and Fms-like tyrosine kinase-1 (FLT-1) in the ulcer tissue. The results indicated that the PDGF and VEGF (*F* = 18.233, *P* < 0.05) mRNA expressions were significantly decreased in the DNIM group compared with the DC group. Jing Wan Hong ointment and rhEGF can increase the mRNA expression of PDGF (*F* = 29.251, *P* < 0.05 or *P* < 0.01). There were significant differences compared with the DNIM group. However, there were no significant differences between all groups for TGF-*β* (*F* = 0.480, *P* > 0.05) and FLT-1 (*F* = 1.141, *P* > 0.05) mRNA expressions. These results are shown in Figure 5.

## 4. Discussion

Several observational studies have shown an even higher mortality rate in patients with DFU, and DFU is an incident with a great impact in an individual's life that also causes a significant burden to the healthcare system and society [5]. In addition, diabetic patients with a DFU history have increased risk of reulceration [6] and lower extremity (LE) amputation as well as higher mortality [7]. Diabetic peripheral neuropathy (DPN) is the most insidious and common and the least understood complication of diabetes. It affects more than 50% of diabetic patients [8]. Neuropathy, peripheral vascular disease, and reduced resistance to infection are recognized risk factors leading to the development of DFUs, which have all the characteristics of a chronic wound [9, 10].

At present, new treatments for diabetic foot ulcer continue to be introduced [11] but, currently, the approved growth factor and cell therapies for diabetic foot ulcers are not routinely used during treatment. Improper wound healing control may result in diabetic foot ulcer or even amputation [12]. Hence, seeking more effective treatments is of major importance. Traditional Chinese medicine (TCM) is widely practiced nowadays and is viewed as an alternative to conventional medicine in different diseases.

Jing Wan Hong ointment has been described as having blood-activating effects based on the traditional practice of Chinese medicine. It can facilitate the healing of ulcers through the improvement of blood circulation to dissipate blood stasis, clearing heat, eliminating dampness, and reducing swelling by detoxification. This study aimed to evaluate the regenerative properties of Jing Wan Hong ointment in healing neurodegenerative type diabetic foot ulcers and investigated the mechanism of action. Our results indicate that Jing Wan Hong ointment had an evident effect on diabetic foot ulcers induced by peripheral nerve injury. The ulcers were decreased in size or healed after 21 days of treatment. The Wagner scores were significantly decreased in the JWH group on day 14 after seven days of administration, as was the case in the rhEGF group. However, these analyses performed for nerve injury ulcer were relatively small (median size, 2-3 cm). It is not clear whether they can be extrapolated to more advanced, larger lesions.

The biological mechanism of wound healing is remarkably similar in almost all types of tissues [13] despite the differences in the type of injury and the organ involved. As various studies indicated, the wound healing process starts with inflammatory responses followed by the generation of new tissue and granulation, recruitment, and growth of endothelial cells for angiogenesis. PDGF, VEGF and its receptor (FLT-1), and TGF-*β* play critical roles in these courses [14–17]. For example, PDGF acts as a potent chemoattractant for mesenchymal cells (including fibroblast cells). It also promotes angiogenesis and upregulates VEGF leading to increased vascularization in an experimental study. VEGF is a key stimulator of angiogenesis that acts to induce mitosis and migration of vascular endothelial cells and its receptor (Flt-1) has tyrosine kinase activity and functions in the opening of Ca^2+^ channels located in the membranes of endothelial cells. TGF-*β* isoforms are profibrotic cytokines, par excellence, and have complex, multifunctional effects on many systems, leading to increased deposition of extracellular matrix (ECM) components (mainly Collagen I) and excessive accumulation of fibrous tissue, having some value mechanisms of wound healing [17, 18]. Therefore, in this study, we performed RT-PCR analysis to detect the mRNA expression of PDGF, VEGF, FLT-1, and TGF-*β* to investigate the action and mechanisms of ulcer healing effect of Jing Wan Hong ointment in nerve injured DFUs. The results showed that Jing Wan Hong ointment increased the mRNA expression of PDGF. However, it did not affect the mRNA expression of TGF-*β*, VEGF, and FLT-1. It has been reported that PDGF has important functions during wound healing and it plays a role in almost all stages of the wound healing process [19]. PDGF can accelerate healing [20, 21], by improving inflammation [22], cell proliferation [23], and angiogenesis [24, 25], and may have contributed to tissue remodeling [19] in diabetic and nondiabetic wound models. A recent study revealed that PDGF administration may contribute to an increase in wound tissue antioxidant capacity [25]. Our results verified, for the first time, that Jing Wan Hong ointment was effective for treatment of nerve injured DFUs rats through tissue regeneration, angiogenesis, and inflammation control. So we speculate that the healing effect of Jing Wan Hong ointment on nerve injured DFUs in rats seemed to be dependent on increasing mRNA expression of PDGF, whose value was as much as the positive control (rhEGF) group. However, there were no significant effects on mRNA expressions of TGF-*β*, VEGF, and FLT-1. These effects seemed to be independent of blood glucose control. Nevertheless, this study provides a scientific basis to support the traditional use of Jing Wan Hong ointment in diabetic foot ulcers.

## Figures and Tables

**Table 1:** Primers for PCR.

Gene	Primers	DNA bases	Anneal temperature
GAPDH	Forward: CCAAGGTCATCCATGACAA	579 bp	55.0°C
	Reverse: TGTCATACCAGGAAATGAGC		
PDGF	Forward: CCTGTGCCCATCCGCAGGAAGAGA	227 bp	65.9°C
	Reverse: TTGGCCACCTTGACGCTGCGGTG		
TGF-*β*	Forward: AGTGGATCCACGAGCCCAA	233 bp	51.4°C
	Reverse: AGGAGCGCACGATCATGTT		
VEGF	Forward: CTGCTCTCTTGGGTGCACTGG	320 bp	62.3°C
	Reverse: GGTTTGATCCGCATGATCTGCAT		
FLT-1	Forward: CAAGGGACTCTACACTTGTC	240 bp	53.1°C
	Reverse: CCGAATAGCGAGCAGATTTC		

**Table 2:** The results of the Mann-Whitney Rank Sum Test of the Wagner classification (*n* = 10).

Groups	DC					DNIM				JWH				rhEGF			
Days after administration		7	14	21	28	7	14	21	28	7	14	21	28	7	14	21	28
Compared with DC	*z*	—	—	—	—	−3.775	−4.069	−4.033	−3.706	−3.446	−4.067	−3.410	−2.781	−4.147	−4.051	−3.430	−2.450
	*P*	—	—	—	—	0.000^*^	0.000^*^	0.000^*^	0.000^*^	0.001^*^	0.000^*^	0.001^*^	0.005^*^	0.000^*^	0.000^*^	0.001^*^	0.014^*^

Compared with DNIM	*z*	−3.775	−4.069	−4.033	−3.706	—	—	—	—	−0.576	−2.317	−3.029	−3.463	−0.559	−2.636	−3.225	−3.495
	*P*	0.000^&&^	0.000^&&^	0.000^&&^	0.000^&&^	—	—	—	—	0.565	0.021^&^	0.002^&&^	0.001^&&^	0.576	0.008^&&^	0.001^&&^	0.000^&&^

Note: “—” denotes no comparison in the two groups of the corresponding line and column in the table. ^*^ *P* < 0.01 versus DC group; ^&^ *P* < 0.05 versus DNIM group; ^&&^ *P* < 0.01 versus DNIM group.
